# A Rare Case of Methotrexate-Induced Gastric Ulcer

**DOI:** 10.7759/cureus.36321

**Published:** 2023-03-17

**Authors:** Kyaw Min Tun, Tooba Laeeq, Salman Mohammed, Katrina Naik, Gordon Ohning

**Affiliations:** 1 Department of Internal Medicine, Kirk Kerkorian School of Medicine at the University of Nevada Las Vegas (UNLV), Las Vegas, USA; 2 Division of Gastroenterology and Hepatology, Department of Internal Medicine, Kirk Kerkorian School of Medicine at the University of Nevada Las Vegas (UNLV), Las Vegas, USA

**Keywords:** rheumatoid arthritis, side effect, proton pump inhibitor, peptic ulcer disease, methotrexate

## Abstract

Methotrexate is commonly used to treat autoimmune conditions and malignancy. Peptic ulcer disease is a sparsely documented side effect of methotrexate. A 70-year-old female patient with rheumatoid arthritis on methotrexate presented with generalized fatigue and was found to be anemic. Endoscopy revealed gastric ulcers, the etiology of which was attributed to methotrexate use after careful exclusion of other possible causes. Cessation of methotrexate has been reported in the literature as vital to the healing of ulcers. Proton pump inhibitors or histamine 2 receptor (H2R) blockers may also be used as treatment; however, methotrexate should be discontinued before initiation of proton pump inhibitors, which can hinder the metabolism of methotrexate and can, in turn, lead to a worsening of the peptic ulcer disease.

## Introduction

Methotrexate is a disease-modifying antirheumatic drug (DMARD) that is widely used in patients with autoimmune conditions such as rheumatoid arthritis and psoriasis [1]. It can also be used to treat conditions such as ectopic pregnancy or malignancy. It functions as a purine synthesis inhibitor [2]. Methotrexate has been linked to a spectrum of adverse reactions and complications. These range from abdominal pain, stomatitis, and nausea to hepatotoxicity, pulmonary toxicity, and myelosuppression. Peptic ulcer disease is a rare side effect of methotrexate that has been sparsely reported in the literature [1,3]. Herein, we report a case of a newly diagnosed gastric ulcer in a patient without known common risk factors for peptic ulcer disease except for methotrexate use.

## Case presentation

**Figure 1:**
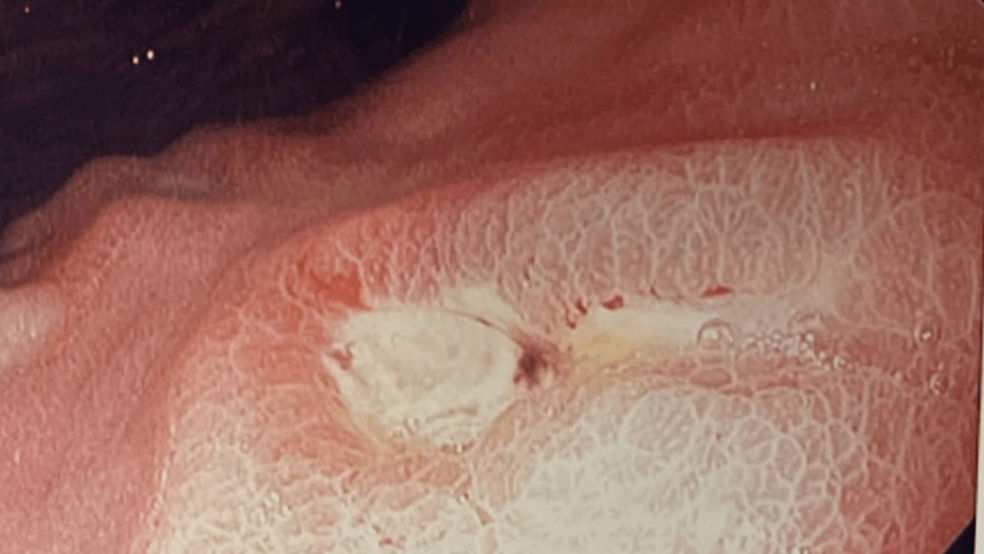
An example of a Forrest III ulcer that was discovered during esophagogastroduodenoscopy. The ulcer had heaped margins and was found in the midgastric body and across the incisura without stigmata of active bleeding. All ulcers were found to be smaller than 2 cm.

A 70-year-old female with a past medical history of rheumatoid arthritis, diabetes mellitus, and hypothyroidism presented with generalized fatigue that had been ongoing for approximately a week. She had recently returned from Mexico. The patient’s hemoglobin on admission was 7.8 g/dL with a mean corpuscular volume (MCV) of 68.8 fL. The patient denied a history of anemia, recent trauma, or external wounds; however, laboratory evaluation was significant for iron deficiency. The following day, her hemoglobin level further declined to 5.5 g/dL and required transfusion, which improved the hemoglobin to 7.7 g/dL. There was no evidence of bleeding. Esophagogastroduodenoscopy was performed and demonstrated seven Forrest III ulcers with heaped margins in the midgastric body and across the incisura, without stigmata of active bleeding (Figure 1). All the ulcers were noted to be smaller than 2 cm. The rest of the endoscopy was unremarkable. Biopsies taken from the ulcers revealed fibrinopurulent debris and slight atypia in the glandular epithelium, consistent with intestinal metaplasia. Biopsies of the gastric antral mucosa showed focal surface erosions with reactive changes without evidence of* Helicobacter pylori*, Epstein-Barr virus (EBV), or cytomegalovirus (CMV) infection.

According to the patient, she had no recent flare-ups of rheumatoid arthritis. Her symptoms were well-controlled with methotrexate, and she had not required nonsteroidal anti-inflammatory drugs (NSAIDs) or steroids for pain for at least two years. The patient indicated that methotrexate was started two years ago by her rheumatologist. However, she had not been evaluated by a healthcare professional until this admission. The patient denied a history of tobacco dependence, alcohol dependence, or a family history of peptic ulcer disease. Besides methotrexate 25 mg per week, levothyroxine, and metformin, the patient took no other medications, including NSAIDs and folic acid. After a thorough review of the current literature and careful exclusion of other possible causes of gastric ulcers, including stress-related causes, it was determined that methotrexate was associated with the development of the ulcer. Consequently, DMARD was discontinued, and oral pantoprazole and oral iron supplements were initiated thereafter. The patient was advised to follow up with a rheumatologist and was discharged. Two months after hospitalization, the patient remained off methotrexate without exacerbation of rheumatoid arthritis symptoms. The patient continued taking oral pantoprazole daily and oral iron supplementation. She had been started on hydroxychloroquine during that time and had not experienced flare-ups. The patient’s generalized fatigue had resolved; she denied symptoms such as abdominal pain, melena, coffee-ground emesis, hematemesis, and hematochezia. The patient was also no longer anemic, with hemoglobin and MCV 12 g/dL and 85.7 fL, respectively.

## Discussion

While oral mucositis or oral mucocutaneous side effects have been linked to methotrexate, gastric ulcers are rare adverse events associated with DMARD. The incidence has only been sparsely documented in the literature. In an observational study by van Dooren-Greebe et al. [1], three patients developed gastric ulcers due to methotrexate use among a cohort of 113 patients. In those patients, temporary cessation of methotrexate was required for the gastric ulcers to resolve [1]. A study by Sartori et al. [4] also reported the impact of different types of chemotherapy, including methotrexate, on the gastrointestinal mucosa. It was determined that methotrexate was one of the most injurious chemotherapy regimens for upper gastrointestinal mucosa [4].

Treatment for methotrexate-induced gastric ulcer includes stopping the offending agent and using proton pump inhibitors (PPI) or histamine 2 receptor (H2R) blockers [3-6]. However, it is crucial to discontinue methotrexate before the initiation of PPI and consider alternative therapies such as hydroxychloroquine. Renal excretion of methotrexate includes the uptake of the methotrexate compound via human organic anion transporter 3 (hOAT3) into renal proximal tubule cells [6]. PPIs, especially lansoprazole, esomeprazole, rabeprazole, and omeprazole, have been demonstrated to inhibit hOAT3, which, in turn, decreases the metabolism of methotrexate and can lead to the worsening of side effects, including gastric ulcers [6]. Therefore, methotrexate should be discontinued before starting PPI. On the other hand, H2R blockers such as famotidine or ranitidine have not displayed an inhibitory effect on hOAT3 or methotrexate metabolism [6].

 Methotrexate has also been noted to interfere with the healing of existing gastric ulcers. In a case report by Kiyotoki et al., only after discontinuation of methotrexate was gastric ulcer healing demonstrated in a patient who had otherwise continued to experience delayed healing despite medical and endoscopic treatment [3]. The mechanism of injury is unclear. It is believed that the mechanism could be related to folate antagonism and subsequent inhibition of DNA synthesis [3]. The threshold dosage of methotrexate associated with an increased risk of gastric ulcer, if there is such a limit, has not been reported in the literature at the present. In clinical application, however, before attributing a gastric ulcer to methotrexate, other possible causes ought to be thoroughly evaluated and excluded. These include, but are not limited to, NSAID use, tobacco dependence, alcohol dependence, family history, gastric cancer, CMV, EBV, and *H. pylori* infections.

## Conclusions

Overall, methotrexate-induced peptic ulcers have only been sparsely documented in the literature. Methotrexate should be considered in the differential diagnosis of any patient with endoscopically proven gastric ulcers without a clear etiology. Cessation of methotrexate may be considered if the patient otherwise does not have rheumatoid arthritis-related symptoms and requires patient-centered coordination between multiple disciplines.
